# Low to moderate-dose off-label use of second-generation antipsychotics seems to be safe in the light of cardiac adverse effects in child psychiatric patients

**DOI:** 10.1007/s00431-025-06447-4

**Published:** 2025-09-11

**Authors:** Kirsi Kakko, Raili Salmelin, Kaija Puura, Tuija Poutanen

**Affiliations:** 1 Faculty of Medicine and Health Technology, Tampere University, Tampere, Finland; 2 Department of Child Psychiatry, Tampere University Hospital, Wellbeing Services County of Pirkanmaa, Tampere, Finland; 3 Department of Paediatrics, Tampere University Hospital, Wellbeing Services County of Pirkanmaa, Tampere, Finland

**Keywords:** Antipsychotic, Child, ECG, QT interval, Cardiac adverse effect

## Abstract

Second-generation antipsychotics (SGAs) may increase the risk for ventricular arrhythmias by prolonging the rate-corrected QT interval (QTc) on an electrocardiogram (ECG). This prospective study aimed to examine QTc changes in child psychiatric patients during the implementation of an SGA monitoring protocol. QTc was calculated using both Bazett’s (QTcB) and Fridericia’s (QTcF) formula and categorised as normal (≤ 450 ms), borderline (> 450, < 470 ms), prolonged (≥ 470, < 500 ms), or significantly prolonged (≥ 500 ms). The study patients (n = 55, 76% males, median age 9.9 years) were followed for a median of 9 months. In all of them SGA (risperidone, aripiprazole, quetiapine) treatment was off-label. The median olanzapine equivalent dose was 2.0 mg. Concurrent ADHD medication was used by 40%. A baseline (BL) ECG was available for 78%. A BL and at least one follow-up ECG existed for 75%. The mean change in QTcB was 18.1 ms (*p* < *0.001*) and in QTcF 17.2 ms (*p* < *0.001*). The QTcB change was significantly greater in females (31.7 ms vs 13.2 ms, *p* = *0.046*). During the follow-up, 11% had borderline (n = 5) or significant (n = 1) QTcB prolongation. In 5/6, the prolongation appeared after a change in psychotropic medication. No arrhythmias were detected.

*Conclusion*: Low to moderate-dose SGA treatment in children seems to be safe in the light of cardiac adverse effects. However, individual risk assessments and systematic ECG monitoring are necessary. Particular attention should be paid to medication changes and female patients.

**Table:** 

**What is Known:** • *Second-generation antipsychotics (SGAs) may prolong the rate-corrected QT interval (QTc) and increase the risk for ventricular arrhythmias.* • *The risk seems to be low in healthy children, but data on long-term treatment and patients with risk factors are scarce.*	
**What is New:** • *In this clinical sample of 55 children using SGAs, QTc prolongation was seen after changes in psychotropic medication.* • *Individual cardiac risk assessment including family history and monitoring of SGA treatments is vital. Attention should be paid to medication changes and female patients.*

**What is Known:**

• *Second-generation antipsychotics (SGAs) may prolong the rate-corrected QT interval (QTc) and increase the risk for ventricular arrhythmias.*

• *The risk seems to be low in healthy children, but data on long-term treatment and patients with risk factors are scarce.*

**What is New:**

• *In this clinical sample of 55 children using SGAs, QTc prolongation was seen after changes in psychotropic medication.*

• *Individual cardiac risk assessment including family history and monitoring of SGA treatments is vital. Attention should be paid to medication changes and female patients.*

## Introduction

Second-generation antipsychotic medications (SGAs) are often cornerstones in the treatment of serious mental disorders, such as schizophrenia and bipolar disorder, in all age groups. In children, SGAs are also used, often off-label, in the treatment of various other mental disorders and symptoms, such as irritability and aggression associated with autism spectrum disorders, attention deficit and hyperactivity disorder (ADHD), and conduct disorders [1–3]. The use of SGAs in children has increased in recent decades [4, 5], and concomitant use with other psychotropics, such as stimulants, is common [2, 6]. Due to the nature of the indications, symptomatic SGA treatment may be needed for a long time.

SGAs have a marked potency to induce especially metabolic adverse effects [7]. Cardiac effects, most typically the prolongation of the rate-corrected QT interval (QTc) are also known [8, 9]. SGAs may also associate with orthostatic hypotension and tachycardia [10]. Prolonged QTc, especially when exceeding 500 ms, may indicate a risk of ventricular arrhythmias and sudden death [9, 11, 12]. The current literature on the effects of SGA on QTc in paediatric patients is limited, but the risk seems to vary by SGA and the dosage, with ziprasidone and risperidone having the highest and aripiprazole the lowest risk [8, 9, 11]. Also other psychotropics, such as antidepressants and stimulants, and polypharmacy may increase the risk for QTc prolongation [13, 14]. Individual-related risk factors that may contribute to the risk include: genetic predispositions, e.g. familial long QT-syndrome (LQTS) or a family history of sudden cardiac deaths, female gender, structural cardiac abnormalities, electrolyte imbalance, excess weight or previously prolonged QTc [8, 12, 15]. Further, symptoms such as syncope or exercise-induced events may indicate an individual risk. With individual predisposition, QTc prolongation may occur independently of the dosage [11]. Studies show that the risk of pathological QTc prolongation during SGA treatment appears to be low in healthy children [8, 9, 14, 15]. However, most studies focus on short-term treatment and often exclude patients with potential cardiac risk factors, such as elevated baseline QTc or concurrent psychotropic use [9]. In clinical practice, the risk must be individually assessed. Further, sometimes, treatment may be necessary despite the presence of potential risk factors. The aim of this study was to examine changes in ECG parameters, particularly in QTc, among a prospective clinical sample of children who were systematically monitored during SGA treatment.

## Materials and methods

This study was conducted at the child psychiatric department of Tampere University Hospital (TAUH) between 15.1.2015 and 31.1.2019 and was a part of a prospective investigation performed during the implementation of a systematic SGA monitoring protocol [16]. During the study period, attending physicians informed patients and guardians about the possibility to enter the study when SGA medication was initiated or changed. The inclusion criteria was a maximum age of 13.0 years and at least one ECG recording during the monitoring period. Data for study purposes were collected from 1) the monitoring forms filled by the physicians [16], and 2) ECG recordings. The ECG monitoring was scheduled: 1) at the baseline (BL), 2) at one month, 3) at 4 months, 4) at 10 months post-BL, and 5) every 6 months thereafter. The duration of follow-up was calculated from BL to the last reported follow-up visit. All participants received standard child psychiatric care (both outpatient and inpatient care when needed). The study protocol, detailed elsewhere [16–18], guided only the SGA monitoring. The choice of medication or other treatments was not influenced. During follow-up the ECG was interpreted by attending child psychiatrist. A pediatric cardiologist was consulted in case of abnormal findings. For the BL recording, an ECG was considered eligible if taken within one week of initiating risperidone or quetiapine, or within two weeks of initiating aripiprazole. Timeframes were based on the median SGA doses prescribed at BL (0.25 mg for risperidone, 2.5 mg for aripiprazole, anfvd 25 mg for quetiapine) and on their pharmacodynamic profiles. To enable dose comparisons across different SGAs used by the study patients, doses were standardised using olanzapine equivalents: 1 mg olanzapine corresponded to 1.4 mg aripiprazole, 0.4 mg risperidone, and 32.3 mg quetiapine [19]. For each patient, the mean olanzapine dose across the follow-up, simply referred to as olanzapine dose, was calculated, and the maximum dose identified.

**Fig. 1:**
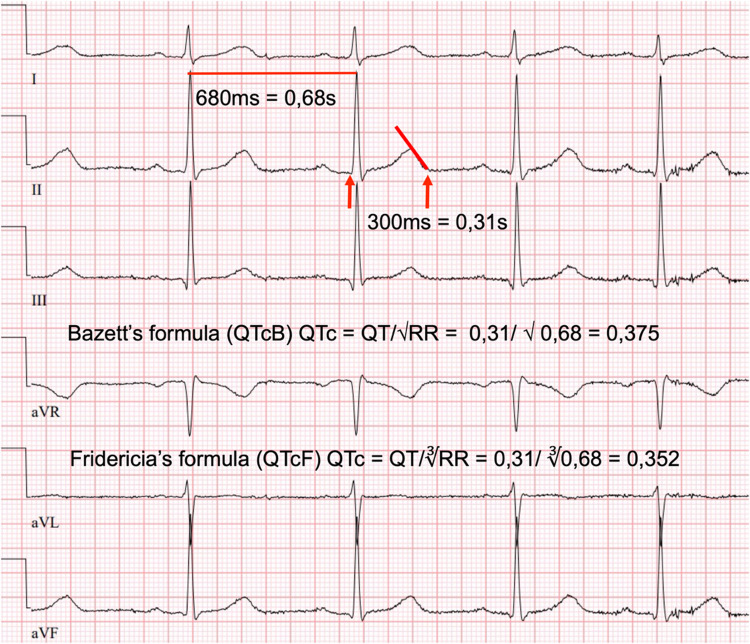
Calculation of rate corrected QT interval (QTc). ECG of an 8-year-old child with a heart rate of 106/min. QTc is measured from the beginning of the Q wave to the end of the T wave. Due to mild respiratory arrhythmia, the longest RR interval has been used in the calculation. The QTc interval has been calculated using both Bazett’s and Fridericia’s formula

Standard 12-lead ECG recordings for the study purposes were analysed by a paediatric cardiologist (T.P.). The QT interval was measured from the beginning of the QRS complex to the end of the T wave. RR represents the interval between two preceding R-waves (Fig. 1). According to Finnish clinical practices QTc was calculated using Bazett’s formula (QTcB) $\left(QTc=\frac{QT}{\sqrt{RR}}\right)$ [20]. As the Bazett’s formula may overestimate QTc particularly at lower and higher heart rates, also Fridericia’s formula (QTcF) $\left(QTc=\frac{QT}{\sqrt[3]{RR}})\right)$ was used for the study purposes [20, 21]. QTc values were categorised as follows: QTc ≤ 450 ms was considered normal [22], > 450 but < 470 ms borderline prolonged, ≥ 470 but < 500 ms prolonged, and QTc ≥ 500 ms significantly prolonged. Due to the age of the patients (maximum of 13 years) and the proportion of male patients, the categorisation was used irrespective of gender, even though QTc reference values are considered higher for adolescent females. HR, PQ, and QTcB values were compared to the age and gender-specific standards for children and adolescents [22]. For patients with borderline or prolonged QTcB, psychotropic medication prescriptions, dose changes, and their timely association with observed QTc elevation were investigated. Due to the small sample size, this information was reported in general level. The standardised body mass index adjusted for age and gender (zBMI) was calculated using the Finnish national growth reference tables to identify possible overweight and obesity [23]. Informed written consent was obtained from all patients aged 7 years or older and from their legal guardians. The study was approved by the Ethics Committee of the Pirkanmaa Hospital District, Tampere, Finland.

Categorised variables are reported as frequencies. For normally distributed continuous variables, means (M) and standard deviations (SD) are reported, and for non-normally distributed ones, medians and quartiles (Md, Q_1_, Q_3_) are reported. To test the statistical significance of differences between groups, the two-sample t-test was used. The one-sample t-test was applied to examine whether the change in QTc time differed statistically significantly from zero. *P*-values less than 0.05 were considered significant and 0.05–0.1 as indicative. SPSS Statistics, version 29 (IBM Corporation, New York, USA) was used for all the statistical analyses.

## Results

The mean age of the study participants (n = 55, 76% males) was 9.9 years (SD 1.8, min 5.9, max 13). The median follow-up time was 9 months (Q_1_ 3.6, Q_3_ 19.1, min 0.5, max 33). The background information of the patients and information on SGA and other psychotropic medications are presented in Table 1. A diagnosis of a hyperkinetic disorder was indicatively more common, and a diagnosis of depression indicatively less common in males compared to females (57% vs. 23%, *p* = *0.055* and 0% vs. 15%, *p* = *0.053*, respectively). No other significant gender differences were seen in the age, background, treatment duration or medication related factors. The family history for cardiac abnormalities was systematically assessed only in the twelve patients (22%) who were referred for cardiology consultation. None of them had cardiac risk factors in the family. At BL, 75% of the patients were antipsychotic naïve. Risperidone was the most commonly initiated SGA, none of the patients used ziprasidone. During the monitoring period, 35% had SGA changed. All prescriptions were off-label, due to the indication or the duration of use. The median olanzapine equivalent dose at BL (n = 52) was 1.0 mg (min 1.0, max 2.0) and during the study period 2.0 mg (Q_1_ 1.0, Q_3_ 2.6, min 1.0, max 6.5). There were no statistically significant gender differences in the median doses (Md 2.0, Q_1_ 1.2, Q_3_ 2.6, min 1.0, max 6.5 in males vs. Md 2.0, Q_1_ 1.0 mg, Q_3_ 2.4, min 1.0, max 3.5 in females, p = *0.416*) during the study period. A concurrent ADHD medication was used by 40% at some point of the monitoring period (Table 1).

**Table 1:** Patient and SGA medication-related characteristics among the study patients

	Boys (*n* = 42)	Girls (n = 13)	*p*	All (n = 55)
	%	%		%
Diagnosis (ICD-10 class)				
Hyperkinetic disorders (F90)	57	23	*0.055*	49
Conduct/Mixed conduct and emotional disorders (F91-92)	50	31	*0.341*	46
Developmental disorders (F83-84)	19	15	*1.000*	18
Other psychiatric diagnosis (F20-43, F93-95)^a^	0–10	0–15	*0.053–1.000*	2–9
Indication for SGA initiation				
Aggression and conduct problems	64	46	*0.334*	60
Mood dysregulation	43	39	*1.000*	42
Psychosis or hallucinations/delusions	19	31	*0.448*	22
Anxiety/fears	12	23	*0.376*	15
Self-destructive behaviour	12	15	*1.000*	13
SGA status at baseline			*0.212*	
Naïve	69	92		75
Switching SGA	19	0		15
Unclear	12	8		11
Initiation SGA			*0.138*	
Risperidone	52	77		58
Aripiprazole	36	8		29
Quetiapine	12	15		13
SGA used at some point of the follow-up				
Risperidone	60	77	*0.333*	64
Aripiprazole	60	39	*0.216*	55
Quetiapine	17	15	*1.000*	16
Other psychotropic medication^b^				
ADHD medication	45	23	*0.204*	40
SSRI	10	23	*0.337*	13
Benzodiazepines	2	0	*1.000*	2
Melatonin	55	23	*0.060*	47

^a^Diagnoses with frequencies less than 10% in the whole sample are not specified individually

^b^At least a trial during the study period

**Fig. 2:**
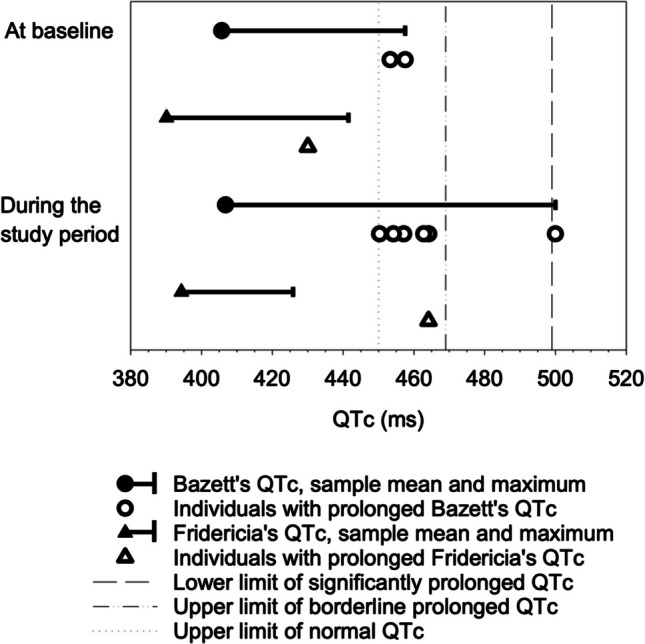
QTc mean and maximum values in a sample on child psychiatric patients (n = 55) as well as the QTc values of individuals with prolonged QTc. Limits of normal QTc, borderline and significantly prolonged QTc values are also shown

Information on the ECG recordings-related frequencies are reported in Table 2. ECG parameters at BL and across the study period are reported in Table 3 and Fig. 2. A Both BL ECG, and BL and at least one other ECG was recorded in three quarters of the patients, the latter allowing the examination of changes in ECG parameters. No statistically significant gender differences were seen in the BL QTcB (M 404.9 ms, SD 26.7, min 360.7, max 453.3 in males vs. M 408.0 ms, SD 31.3, min 346.4, max 457.5 in females, *p* = *0.744*) or QTcF (M 389.7 ms, SD 20.4, min 356.2, max 441.5 in males vs. M 391.2 ms, SD 24.8, min 350.9, max 430.0 in females, *p* = *0.844*). HR deviated from the reference values very seldom. A prolonged PQ was rare, as well, and none had a PQ interval over 200 ms. During the study period QTcB exceeded the reference value for prolongation more frequently than QTcF (Table 2).

**Table 2:** Electrocardiogram (ECG)-related characteristics in child psychiatric study patients (n = 55)

	%
ECG registered	
At baseline	78
At least twice during the study period	95
At baseline and at least once during the study period	75
Non-normative HR^a, b^	
At baseline	0
During the study period (max value)	2
Non-normative PQ^a, b^	
At baseline	6
During the study period (max value)	16
Borderline prolonged QTc^c^, Bazett’s formula	
At baseline	4
At least once during the study period	9
Significantly prolonged QTc^d^, Bazett’s formula	
At baseline	0
At least once during the study period	2
Borderline prolonged QTc^c^, Fridericia’s formula	
At baseline	0
At least once during the study period	2
Significantly prolonged QTc^d^, Fridericia’s formula	
At baseline	0
At least once during the study period	0

^a^HR = heart rate,

PQ = the interval between the beginning of the P wave and the Q wave

QTc = the rate-corrected QT interval on an ECG

^b^For age and gender

^c^Above 450 but below 470 ms

^d^500 ms or higher

**Table 3:** Electrocardiogram (ECG) parameters in child psychiatric study patients at baseline and during follow-up

Parameter	Baseline (n = 43)				Follow-up (n = 52)				
					Mean across all follow-up ECG recordings				Overall max
	Mean	SD	Min	Max	Mean	SD	Min	Max	
HR^a^ (bpm)	78	14.6	49	114	72.2	10.5	47.8	104.3	119
PQ^a^ (ms)	131.8	19.5	96.0	172.0	138.2	19.1	98.2	191.0	192
QTcB^a, b^ (ms)	405.7	27.7	346.4	457.5	406.8	18.1	362.3	445.0	500
QtcF^a, c^ (ms)	390.1	21.4	350.9	441.5	394.3	15.1	354.6	425.9	464

^a^HR = heart rate,

^a^PQ = the interval between the beginning of the P wave and the Q wave,

^a^QTc = the rate-corrected QT interval on an ECG

^b^Bazett’s formula

^c^Fridericia’s formula

In patients with at least any two ECG recordings (n = 52, 23% females, 35% overweight or obese), the mean QTcB across the study period was indicatively longer in females (M 404.4 ms, SD 18.0, min 362.3, max 440.5 in males vs. M 414.9 ms, SD 16.5, min 381.1, max 445.0 in females, *p* = *0.078*) compared to males. The respective QTcF did not show significant gender differences (M 393.0 ms, SD 15.6, min 354.6, max 425.9 in males vs. 398.5 ms, SD 12.9, min 369.5, max 416.5 in females, *p* = *0.273*). Mean QTcB was indicatively shorter in patients who were considered overweight or obese than those of normal weight (M 401.0 ms, SD 16.8, min 377.4, max 440.5 vs. M 409.8 ms, SD 18.2, min 362.3, max 445.0 vs, *p* = *0.095*). Weight status did not have significant effect in the mean QTcF values (M 390.3 ms, SD 17.7, min 361.3, max 425.9 vs. 396.4 ms, SD 13.3, min 354.6, max 416.5, *p* = *0.166*).

Among patients with both a BL and at least one follow-up ECG available (n = 41, 27% females, 12% quetiapine users, 32% overweight or obese), the mean change in both QTcB and QTcF during the study period was statistically significant (Table 4). In quetiapine users the mean QTcB change was indicatively larger than that of the patients using other SGAs. The mean change in QTcB was statistically significantly larger in females (M 13.2 ms, SD 26.6, min −39.8, max 81.1 in males, vs. M 31.7 ms, SD 22.6, min −10.9, max 73.8 in females, *p* = *0.046*). The respective change in QTcF was not statistically significant (M 14.2 ms, SD 20.8, min −34.6, max 60.4 in males, vs. M 25.3 ms, SD 16.7, min −6.9, max 47.3 in females *p* = *0.118*). Overweight or obesity had no significant effect on the mean change in QTcB or QTcF (*p* = *0.311 and p* = *0.541,* respectively), nor did the concurrent psychotropic use (Table 4). The effect of concurrent SSRI use was not analysed due to the small sample size (n = 4).

**Table 4:** Observed changes in the rate-corrected interval between the beginning of the Q wave and the T wave (QTc) according to the medication used, in child psychiatric study patients who had both baseline and at least one follow-up electrocardiogram available

	QTc change (max – baseline, ms)										
		Bazett’s formula					Fridericia’s formula				
	n	Mean	SD	Min	Max	*p*^b^	Mean	SD	Min	Max	*p*^b^
All	41	18.1	26.6	−39.8	81.1	< *0.001*	17.2	20.2	−34.6	60.4	< *0.001*
Risperidone						*0.224*					*0.227*
Yes	26	22.0	26.5	−18.3	81.1		20.1	19.0	−10.6	60.4	
No	15	11.4	26.4	−39.8	50.5		12.1	21.7	−34.6	45.5	
Aripiprazole						*0.733*					*0.684*
Yes	23	19.4	29.5	−39.8	81.1		18.3	22.2	−34.6	60.4	
No	18	16.5	23.2	−18.3	50.7		15.7	17.7	−10.6	45.5	
Quetiapine						*0.088*					*0.211*
Yes	5	37.2	13.2	18.2	48.5		27.8	11.1	19.7	45.5	
No	36	15.5	27.0	−39.8	81.1		15.7	20.8	−34.6	60.4	
Concurrent ADHD medication^a^						*0.689*					*0.504*
Yes	16	16.0	22.6	−18.3	55.5		19.8	15.7	−6.9	43.6	
No	25	19.5	29.3	−39.8	81.1		15.5	22.7	−34.6	60.4	

^a^Stimulants or atomoxetine

^b^For “All”, deviation from zero, for medications difference between “Yes” and “No” group

In the patient-wise analysis, two patients (4%), had a borderline prolonged QTcB at BL, none had significant prolongation. During the follow-up, six patients (11%) had either borderline (n = 5, 9%) or significantly prolonged (n = 1) QTcB (Table 2). All of these six patients were antipsychotic naïve at BL and underwent several ECG recordings (min 3, max 9) during the follow-up. Five were prescribed risperidone and one quetiapine at BL. Three patients used concomitant psychotropic medications (fluoxetine, methylphenidate, or atomoxetine), and three had their SGA changed during the study. In one patient, the reason for the change was QTc prolongation. In five of the six patients, the prolongation was detected after a change in medication, e.g. a dose increase of SGA, methylphenidate or fluoxetine, or a change in SGA. Patients with borderline QTcB at BL continued to have deviations in during the follow-up. None of the patients had a clinical suspicion of ventricular arrythmias at any point of the study.

## Discussion

In this clinical sample of 55 children receiving modest doses of SGAs, a statistically significant prolongation was observed between BL and the highest follow-up measurement in both QTcB and in QTcF. Although Bazett’s formula is commonly used in clinical practice, it is known to potentially overestimate QTc, particularly when HR is low or high [20, 21]. This tendency was evident also in the current study, as the proportion of patients exceeding the QTc reference values was higher (11%) when calculated with Bazett’s formula compared to Fridericia’s formula (2%). Even though HR in the study patients mainly varied within the age-related reference values, in children, especially with hyperkinetic disorders, HR tends often to be high. Thus, Fridericia’s formula may produce more accurate results in this population. However, despite its potential accuracy, HR related reference values for QTcF are not yet well defined [21]. In the current study, the changes seen in QTc were predominantly clinically insignificant. None of the patients exhibited clinical arrythmias, and QTc prolongation rarely required a change in SGA. These findings align with previous studies suggesting that while the risk of SGA-induced pathological QTc prolongation is low in healthy children, it does occasionally occur [9, 12, 14, 15, 24, 25].

The study sample represents common child psychiatric clinical practices fairly well. As in this study also, prescriptions of psychotropic medications (with the exception of ADHD medication) to children are often off-label, usually due to the indication or the duration of the treatment [2, 3]. As in previous studies concerning prepubertal children [5, 6, 26, 27], most of the patients were boys. SGAs, most commonly risperidone, aripiprazole, and quetiapine, were primarily targeted to the treatment of aggression or difficulties in emotional regulation, often simultaneously with other psychotropics. In an animal model, SGAs have been reported to prolong QT intervals in a dose-dependent manner, with haloperidol and risperidone being the most potent [28]. A large cohort study by Ray et all. (2019) even found that children and adolescents receiving high doses, of antipsychotics had an increased risk for unexpected death [29]. The risk was not elevated with lower doses [29]. In their study, the threshold for high-dose use was defined as 50 mg chlorpromazine equivalents, corresponding to approximately 2,5 mg of olanzapine [29]. In the current study, consistent with the off-label indications, SGA doses were relatively low (corresponding to a median of 2.0 mg of olanzapine), and therefore the dose-dependent effects were not evaluated. Aripiprazole seems to have a lower potency to prolong QTc [9]. In this study however, no significant differences in the QTc of the patients using aripiprazole and other SGAs were detected. Further, contradictory to previous findings [14], in the current study, concurrent use of ADHD or SSRI medications had no significant effects on the QTc.

SGAs may bring out a genetic or other liability for QTc prolongation and cause acquired LQTS, predisposing a risk for ventricular arrhythmia [9, 11, 14, 29]. Previous studies show that female gender seems to associate with longer baseline QTc values and greater delayed-rectifier potassium current responses, possibly indicating a higher risk for arrhythmia [8, 12, 15]. However, the effect of gender on the SGA-induced risk for QTc prolongation in children remains unclear, and studies concerning gender differences are scarce [8, 15]. In the current study, gender had no statistically significant effect on the BL or overall QTc values. However, female gender was associated with a significantly greater SGA-induced change in QTcB, but not in QTcF. Overweight or obesity had no significant impact on QTcB or QTcF change in the present study population. Previous studies concerning the matter have been contradictory, with some studies showing a larger QTc increase in overweight patients [15], and others showing no impact [13]. More studies on the matter are needed.

Pathological QTc prolongation was rare in the study patients. However, those few children who had their QTcB categorised as borderline prolonged at BL continued to have QTc deviations during the follow-up. Closer analyses of prescriptions, doses, and their timely associations showed that significant QTc prolongation was in all cases but one associated with a psychotropic alteration: a change or a dose increase of SGA or other concomitant medication. However, a change in SGA was required in only one patient due to QTc prolongation.

The clinical nature of the study, small sample size and the different adverse effect profiles of the SGAs somewhat limit the generalisability of the results. As the information was obtained from the study monitoring forms, clinically relevant details, e.g. changes in medication doses between the monitoring visits or individual or family related cardiac risk factors may have been underreported. A detailed family history was available only for patients who underwent pediatric cardiology consultation. Further, electrolyte imbalances (e.g. hypokalemia and hypocalcemia) which can contribute to the QTc prolongation, were not systematically monitored and may have influenced the results. In order to detect susceptibility, thorough baseline assessment and monitoring practices are of the utmost importance. Individual, family and treatment-related factors need to be considered [8, 11, 14].

## Conclusions

In this prospective clinical cohort of children receiving modest doses of SGAs, the treatment appeared to be safe in the light of cardiac adverse effects. Nevertheless, both borderline and significant QTc prolongation were observed when assessed using Bazett’s formula. SGA induced changes were evident also when assessed using Fridericia’s formula, but remained less significant. More studies with larger clinical samples are needed. The results of this study indicate the need for systematic ECG monitoring in addition to the thorough assessment of individual and treatment-related factors and detailed family history. Special attention should be paid to situations where the medication is altered or augmented with other psychotropics, with particular regard to female patients. At these points, more frequent ECG monitoring is recommended. As with any medications, potential risks need to be balanced with the potential benefits for the patient [30]. The importance of monitoring and individual risk–benefit assessments are highlighted with off-label treatments.

## Data Availability

No datasets were generated or analysed during the current study.

## Abbreviations

ADHD: Attention deficit and hyperactivity disorder
BL: Baseline visit
ECG: Electrocardiogram
LQTS: Long QT-syndrome
Md: Median
M: Mean
QTc: Rate-corrected QT interval
Q_1_,Q_3_: Quartiles
QTcB: QTc calculated using Bazett’s formula
QTcF: QTc calculated using Fridericia’s formula
SGA: Second-generation antipsychotic medications
SD: Standard deviation
SSRI: Selective serotonin reuptake inhibitor
