# Correction to: Low to moderate-dose off-label use of second-generation antipsychotics seems to be safe in the light of cardiac adverse effects in child psychiatric patients

**DOI:** 10.1007/s00431-025-06564-0

**Published:** 2025-10-15

**Authors:** Kirsi Kakko, Raili Salmelin, Kaija Puura, Tuija Poutanen

**Affiliations:** 1 Faculty of Medicine and Health Technology, Tampere University, Tampere, Finland; 2 Department of Child Psychiatry, Tampere University Hospital, Wellbeing Services County of Pirkanmaa, Tampere, Finland; 3 Department of Paediatrics, Tampere University Hospital, Wellbeing Services County of Pirkanmaa, Tampere, Finland

**Correction to: European Journal of Pediatrics (2025) 184:609**

10.1007/s00431-025-06447-4

It has come to our attention that an error occurred in the author names section of the above-mentioned article. The published author list incorrectly presented the family name before the first name for all authors.

The incorrect author list appeared as: Kakko Kirsi · Salmelin Raili · Puura Kaija · Poutanen Tuija.

The correct author list should read: Kirsi Kakko · Raili Salmelin · Kaija Puura · Tuija Poutanen.

Consequently, the article is currently cited as 'Kirsi et al.' instead of the correct 'Kakko et al.'

The publisher sincerely apologizes for this error and any inconvenience it may have caused to the authors and readers.

The original article has been corrected.

